# Breathing patterns and associated cardiovascular changes in intermittently breathing animals: (Partially) correcting a semantic quagmire

**DOI:** 10.1113/EP091784

**Published:** 2024-03-19

**Authors:** Warren Burggren, Andreas Fahlman, William Milsom

**Affiliations:** ^1^ Developmental Integrative Biology Group, Department of Biological Sciences University of North Texas Denton Texas USA; ^2^ Fundación Oceanogràfic Valencia Spain; ^3^ Kolmården Wildlife Park Kolmården Sweden; ^4^ IFM Linkoping University Linkoping Sweden; ^5^ Department of Zoology University of British Columbia Vancouver British Columbia Canada

**Keywords:** apnoea, bradycardia, breath‐holding, diving, heart, lungs, tachycardia, ventilation

## Abstract

Many animal species do not breathe in a continuous, rhythmic fashion, but rather display a variety of breathing patterns characterized by prolonged periods between breaths (inter‐breath intervals), during which the heart continues to beat. Examples of intermittent breathing abound across the animal kingdom, from crustaceans to cetaceans. With respect to human physiology, intermittent breathing—also termed ‘periodic’ or ‘episodic’ breathing—is associated with a variety of pathologies. Cardiovascular phenomena associated with intermittent breathing in diving species have been termed ‘diving bradycardia’, ‘submersion bradycardia’, ‘immersion bradycardia’, ‘ventilation tachycardia’, ‘respiratory sinus arrhythmia’ and so forth. An examination across the literature of terminology applied to these physiological phenomena indicates, unfortunately, no attempt at standardization. This might be viewed as an esoteric semantic problem except for the fact that many of the terms variously used by different authors carry with them implicit or explicit suggestions of underlying physiological mechanisms and even human‐associated pathologies. In this article, we review several phenomena associated with diving and intermittent breathing, indicate the semantic issues arising from the use of each term, and make recommendations for best practice when applying specific terms to particular cardiorespiratory patterns. Ultimately, we emphasize that the biology—not the semantics—is what is important, but also stress that confusion surrounding underlying mechanisms can be avoided by more careful attention to terms describing physiological changes during intermittent breathing and diving.

## Abstract (highlights)

- **What is the topic of this review?**
  This review examines the rather confusing semantics that has been used to describe patterns in the field of cardiorespiratory physiology as it applies to intermittent breathing, particularly in diving species.
- **What advances does it highlight?**
  This review highlights the various cardiorespiratory phenomena associated with intermittent breathing and diving. It highlights the semantic issues associated with describing each and offers a rationale for standardizing terms based on underlying mechanisms to reduce confusion and advance the study of cardiorespiratory phenomena in both medical and comparative physiological investigations.

## 1 INTRODUCTION: WHY THIS ARTICLE?

Ventilation and perfusion are the two convective steps in the oxygen transport cascade (for recent reviews see Clark et al., 2019; Hopkins, 2020; Hopkins & Stickland, 2023; Hsia et al., 2016). Equations describing both ventilation and perfusion processes contain rate components: breathing frequency and heart rate, respectively. Given this, it should not be surprising that both correlate with changes in O_2_ demand (He et al., 2023). Importantly, however, many species do not breathe in a continuous, rhythmic fashion but rather display a variety of breathing patterns that may be characterized by prolonged periods between breaths (‘inter‐breath intervals’), during which the heart continues to beat. Examples of intermittent breathing abound across the animal kingdom (Fahlman et al., 2017; Milsom, 1991; Milsom et al., 2022), including in insects (Bradley, 2006, 2007; Matthews, 2018; Quinlan & Gibbs, 2006; Terblanche & Woods, 2018), crustaceans (McGaw & McMahon, 1998; McMahon, 1999; McMahon & Wilkens, 1977; Wittmann et al., 2012), strictly aquatic fishes (Milsom, 1991; Milsom et al., 2022), air‐breathing fishes (Brauner & Rombough, 2012; Burggren et al., 1986, 2019; Hedrick et al., 1994; Johansen, 1970; Mendez‐Sanchez & Burggren, 2017; Milsom et al., 2022), amphibians (Boutilier & Shelton, 1986; Fonseca et al., 2012; Shelton & Boutilier, 1982; Taylor et al., 2022; Wang et al., 1999; Zena et al., 2017), reptiles (Burggren et al., 2020; Crossley et al., 2016; Taylor et al., 2010), diving birds (Butler & Halsey, 2008; Maina et al., 2010; Williams et al., 2021) and diving and hibernating mammals (Fahlman, 2024; Fahlman et al., 2017; McArthur & Milsom, 1991).

The literature is rich with descriptions of changes in heart rate associated with intermittent breathing—this richness extends to the variety of terms that have been used to describe them. Unfortunately, terms have not been used consistently and/or are only vaguely defined, and often likened to pathological conditions seen in humans, creating confusion as to what each term implies.

We, as authors that have used, and possibly abused, these various terms, undertake here a review of the terminology that has been presented in the literature on intermittent breathing and diving, with suggestions for a possible consensus on the use of terminology. The reader might ask, Why is a review of the terminology of intermittent breathing and diving even necessary? We disagree with the viewpoint of the famous science philosopher Karl Popper who quipped that ‘…we should altogether avoid, like the plague, discussing the meaning of words’ (Popper, 1972). Instead, we take the position of R. H. S. Crossman, who in paraphrasing Socrates, bluntly stated that ‘…if we do not know precisely the meanings of the words we use, we cannot discuss anything profitably’ (Crossman, 1937). Indeed, the biomedical literature is replete with attempts to define terms related to respiratory and cardiovascular function—for a small sampling see (Hobohm et al., 2020; Palatini et al., 2014; Pellegrini & Scheinman, 2016; Powell et al., 2017; Ramirez & Lieske, 2003; Randerath et al., 2017; Spodick, 1993; Yuan et al., 2023). The genesis for our article, then, rests on the following premise: If we do not know precisely what we mean when we and our colleagues use, for example, the terms ‘diving bradycardia’, ‘episodic breathing’ or ‘sinus arrhythmia’, then we put in danger a profitable discussion about specific phenomena. For example, whether classical terms indicating phenomena described in humans are adequate descriptions of the patterns we see in diving vertebrates is contentious, as we shall see below. Moreover, and perhaps more insidiously, behind the terms used in the field of diving physiology are implied mechanism(s) underlying the described phenomenon. Yet, these specific mechanisms may be incorrectly implied, or alternatively ignored, when applying classic terminology to other phenomena.

**FIGURE 1:**
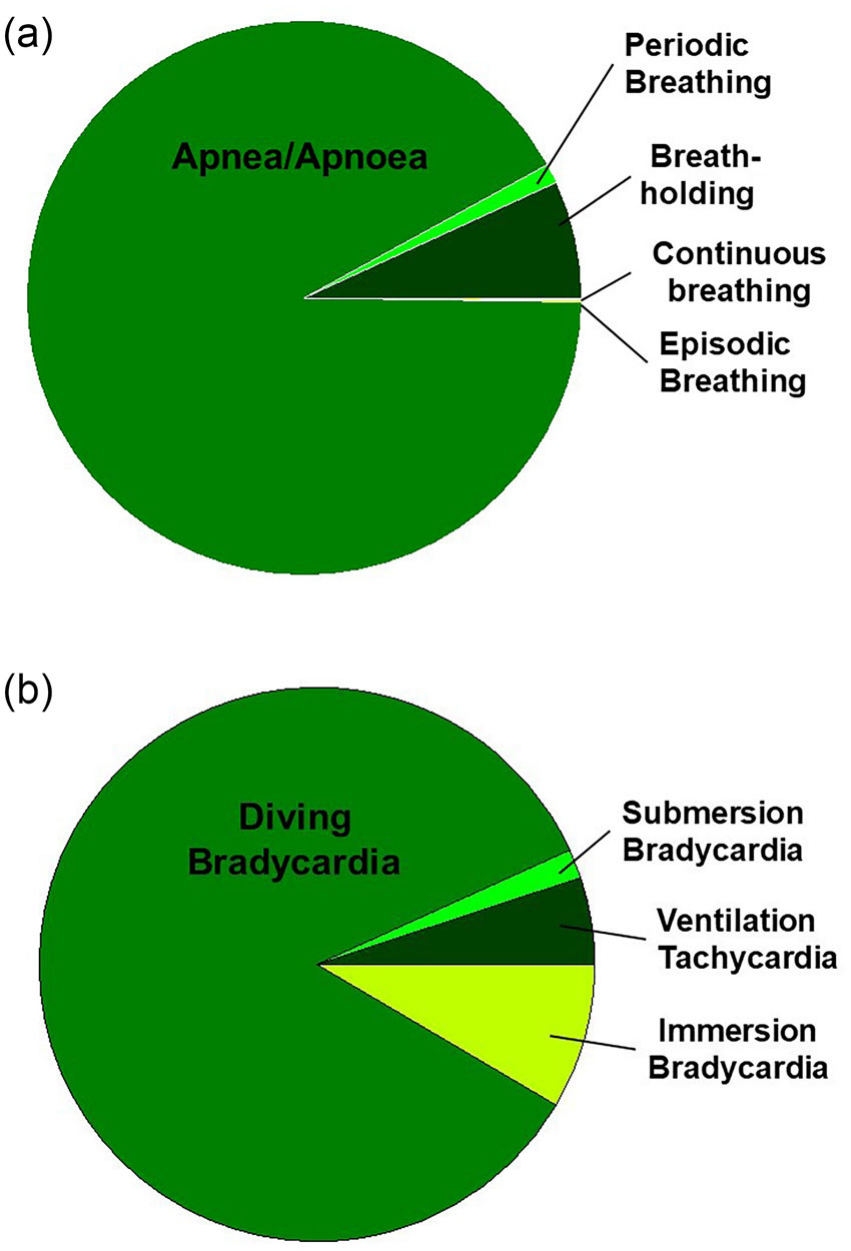
Frequency of occurrence of specified terms associated with (a) apnea/apnoea, and (b) changes in heart rate associated with diving in published journal articles based on PubMed search 11 January 2024.

We follow Crossman's (1937) philosophy of offering precise definitions surrounding the biology of intermittent breathing so that we can precisely discuss associated cardiorespiratory phenomena—in a way that allows the reader to follow our logic flow. To that end, Table 1 provides our own, possibly slightly eccentric, working definitions of respiratory and cardiovascular terms used in this article to describe similar or even identical phenomena associated with intermittent breathing and diving. The proportionate use in the literature of some of these terms is illustrated in a pie chart in Figure 1 and Table 2.

**TABLE 1:** Definitions of terms used or referred to in this article.

Term	Definition (as used in this article)	Example usage of term
Apnoea/apnoeic	Temporary absence of breathing (normal for diving animals, or pathological in otherwise continuously breathing animals)	‘The period of apnea lasted 3 minutes’
Apnoeusis/apnoeustic	An abnormal breathing pattern where expiration is brief and with prolonged gasping inhalation.	‘The breathing pattern was abnormal and apneustic’
Arrhythmic	Lacking rhythm	‘Ventilation was arrhythmic, with no particular pattern evident’
Arrhythmia	Rhythmic variation in heart rate, normal in many vertebrates but also reflecting potential pathology in humans.	‘The sinus arrhythmia was evident during inspiration and exhalation’
Baseline	Starting point used for comparison	‘The baseline heart rate was 70 beat/min, but bradycardia lowered it to 45 beats/min’
Bradycardia	A reduced heart rate, which may be result from normal intermittent breathing or from a pathology	‘A bradycardia was evident during diving’
Breath‐holding	Apnoea either at end‐inspiration or end‐expiration	‘The animal was breath‐holding for 2 minutes before resuming breathing’
Continuous	Occurring without break or interruption	‘Breathing was continuous, without interruption, while the animal was at the water's surface’
Discontinuous	Event occurring with breaks or interruptions (see also Intermittent)	‘Spiracular ventilation discontinuous and irregular’
Diving	Descent below the water's surface	‘The seal continued diving for an average duration of 10 minutes’
Episodic/episode	A group of breaths occurring occasionally at regular or irregular intervals	‘Breathing was episodic because the animal was diving’
Eupnoea	Continuous and regular ventilation of gas exchange organ	‘Eupnea was evident with a breathing frequency of 20 breaths/min’
Immersion /immersed	Act of being under water (see also Submersion)	‘The period of immersion under water was about 3 minutes’
Instantaneous	Measured at a particular instant	‘The instantaneous heart rate was 34 beats/min) at the moment before surfacing’
Inter‐breath	Period between breaths	‘The inter‐breath interval was more than 20 minutes’
Intermittent	Event occurring with breaks or interruptions (see also Discontinuous)	‘Breathing was intermittent even though the animal was at the water's surface for several minutes’
Irregular	Unevenly occurring or unevenly spaced	‘The animal showed irregular periods of diving from 2 up to 20 minutes’
Normal	Non‐pathological, typically exhibited phenomenon; can be a baseline condition	‘The animal showed a normal pattern of heart beat change during intermittent diving’
Periodic	Occurring at intervals of similar or dissimilar length.	‘Breathing was periodic because the animal was intermittently diving’
Resting	At minimal levels of maintenance activity	‘Resting heart rate of the seal on the beach was 85 beats/minute’
Respiratory sinus arrhythmia	Heart rate variation throughout ventilation, with rate acceleration and deceleration during inspiration and expiration, respectively.	‘Heart rate showed a clear respiratory sinus arrhythmia while the animal was breathing at the surface’
Rhythm/rhythmic	Phenomenon occurring in regularly repeating, identifiable rhythm	‘Ventilation in the resting seal was rhythmic, with no periods of apnea’
Submersion/submersed	Act of being under water (see also Immersion)	‘Submersion last ∼30 minutes before the whale returned to the surface’
Tachycardia	An elevated heart rate, which may be a normal response during intermittent breathing or from a pathology	‘A tachycardia occurred upon surfacing’

Some terms have both a common English meaning and a more technical meaning as used by physiologists. We are not attempting to re‐define any term in this table, but merely clarifying how we use them in our discussion.

**TABLE 2:** Frequency of occurrence of specified terms in published journal articles based on PubMed search 11 January 2024.

VENTILATION‐RELATED TERMS	NUMBER OF CITATIONS
**‘**Intermittent breathing’	29
‘Continuous breathing’	111
‘Periodic breathing’	1,000
‘Episodic breathing’	45
‘Apnea’/‘Apnoea’	69,557/10,721
‘Breathholding’/‘Breath‐holding’	1,899/3,962
‘Discontinuous breathing’/‘Discontinuous ventilation’	3/13

CARDIAC‐RELATED TERMS	NUMBER OF CITATIONS
‘Ventilation tachycardia’	6
‘Submersion bradycardia’	2
‘Diving bradycardia’	100
‘Immersion bradycardia’	10

With provided definitions, we now explore the most commonly used terminology in the fields of intermittent breathing and diving biology, and then attempt a synthesis based on our review of the ‘words we use’ (Crossman, 1937). As we do so, however, we want to state an obvious problem—namely, some terms have both a common usage in English vernacular and a clinical meaning. We also note that, while it is tempting to delve more deeply into actual physiological mechanisms, such a review is beyond the scope of the current article. As a remedy, we refer to numerous mechanistically oriented original research articles and reviews with such information.

## 2 THE ‘PROBLEM’ WITH RELATIVITY AND INTERMITTENT BIOLOGICAL PHENOMENA

Before we delve into the semantic ‘quagmire’ alluded to in our article's title, we need to deal with the issue of ‘relativity’, defined by Oxford Languages as ‘the absence of standards of absolute and universal application’. In the field of intermittent breathing and diving, there are several concepts that are relative per se, and are reflected in the terms that we use in our written communications. Examples include ‘normal’, ‘natural’, ‘typical’, and so forth. Thus, for example, when it is said that ‘Cetaceans normally hold their breath’, needless to say no investigator means to imply that it is thus ‘abnormal’ to actually breath. Rather, this refers to that fact that a greater proportion of the cetaceans’ time is spent holding their breath, either during long inter‐breath periods or while underwater. In writing this article (and as astutely pointed out by a referee of this article), many of the commonly used terms refer to quantitative characteristics of a time series. It is tempting to try to assign actual values to chronologically referenced words like ‘periodic’, ‘rhythmic’, ‘intermittent’, ‘continuous’, and so forth, saying for example ‘intermittent’ means a phenomenon interrupted by >2 standard deviations of some mean time period tied to the biology of the animal. The problem of relativity rears its head with this approach. As we discuss below, to use the term ‘intermittent breathing’ to describe an animal spending most of its time breathing at 15 breaths/min but then pausing for two inter‐breath intervals would not similarly be applicable to an animal that breaths regularly and rhythmically at only one breath/minute. Thus, some of the time‐related terms used in the field (and used by us in this article) need to be quantified for each species or group of species.

Relativity is not only a problem mired in time series analyses. Researchers often want to establish a ‘baseline’. If, for example, the resting heart rate in a human subject varies between 70 and 75 beats/min, the baseline for heart rate change is fairly easy to establish. If, however, the heart rate of an intermittently breathing animal in its natural environment varies from 25 to 100 beats/min over a day, then the concept of ‘baseline’ is less useful 2020c. Putting this in a cardiovascular context, the commonly used terms ‘bradycardia’ and ‘tachycardia’, referring to a slower and faster heart rate, respectively, are compared to what baseline? When the heart is at its lowest value? Highest value? Longest steady value?

Our goal is not to solve the problem of physiological relativity in this article, but rather to point out associated semantic problems and then use our own terminology as precisely and carefully as possible.

## 3 COMMONLY USED SCIENTIFIC TERMINOLOGY ASSOCIATED WITH INTERMITTENT BREATHING AND DIVING

### 3.1 Terms used to describe ventilatory patterns

#### 3.1.1 ‘Continuous (resting, rhythmic) breathing’ or ‘eupnoea’

##### The phenomenon

**FIGURE 2:**
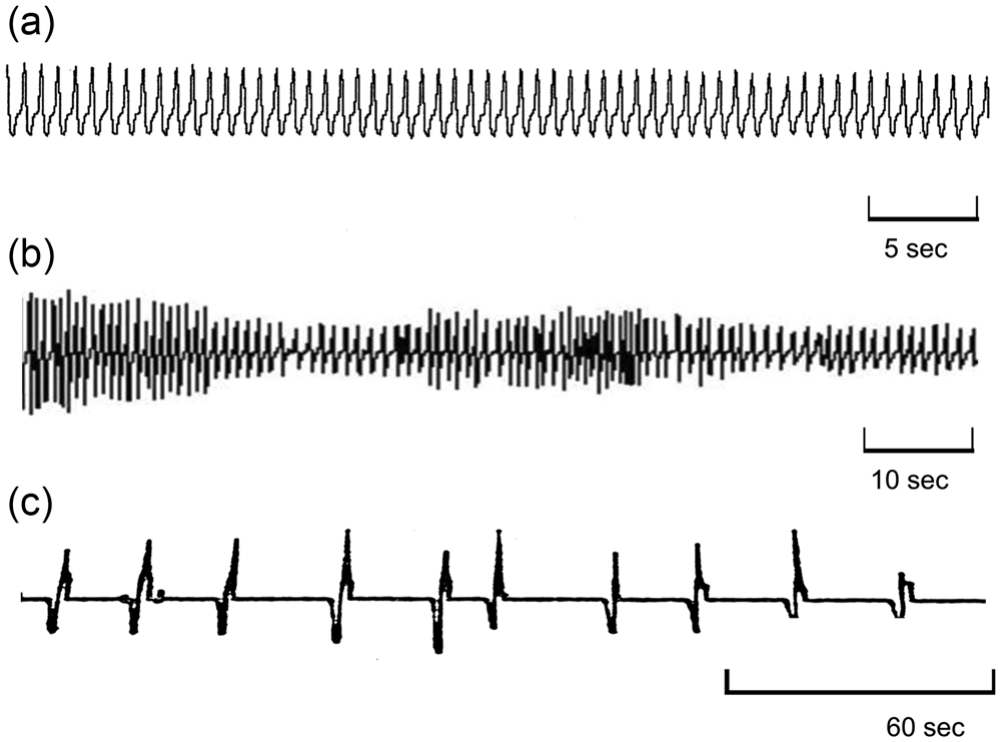
Continuous breathing. Breathing in the neotropical fish tambaqui (*Colossoma macropomum*) showing patterns that are both (a) rhythmic and regular and (b) arrhythmic with sporadic changes in frequency and amplitude (modified from Reid et al., 2003). (c) Continuous breathing in the tortoise (*Testudo graeca*) showing slow continuous breathing. (From W. Burggren, unpublished observation).

The resting, non‐pathological, breathing pattern (i.e., ‘normal’) found in many animals, including birds and mammals, is one of continuous, rhythmic breathing, also termed ‘eupnoea’ (Figure 2a). In terrestrial mammals, each breath consists of three or four phases. At rest, inspiration is followed by a post‐inspiratory phase during which passive expiration occurs, although activity continues to be present in inspiratory muscles serving to brake the expiratory air flow. This is often referred to as the E1 phase (Jenkin & Milsom, 2014). Following is an expiratory phase (E2). Passive expiratory flow may continue in E2 but, when breathing is slow, this phase may also contain an end expiratory pause during which there is no air flow. When respiratory drive is elevated, a fourth phase, active expiration (E3, also called AE), is recruited. This phase usually occurs immediately before inspiration (after the passive expiration). In birds, as well as in reptiles where breathing is continuous (i.e., without irregular apnoeic time intervals between breaths), the cycle begins with an active expiration followed immediately by active inspiration. When breathing is slow in these species, the inspiration may be followed by glottal closure and an end‐inspiratory pause. Note that regular pauses in air flow may occur even in continuous breathing patterns.

Resting eupnoeic breathing patterns are usually *rhythmic*—that is, breaths occurring regularly in an identifiable rhythm. However, breaths may also be *arrhythmic* under various circumstances with irregular inter‐breath intervals and/or tidal volumes (Figure 2b). These latter patterns of breathing are also sometimes referred to as ‘periodic breathing’ (see next section).

Rhythmic, continuous breathing patterns are the resulting output of a series of conditional central rhythm generators found in the medulla of all vertebrates (Milsom et al., 2022). The homology of the various rhythm generating sites in different vertebrate classes is debated, but there is consensus that all require a conditional input, typically related to metabolic rate and the need to acquire O_2_ and/or eliminate CO_2_ (Milsom et al., 2022). These continuous breathing patterns are the result of the rhythmic interaction between the various sites, collectively referred to as the central rhythm generator (CRG) and peripheral sensory inputs.

##### Semantic issues

When the end‐inspiratory pause (birds) or end‐expiratory pause (mammals) is less than a second, the term ‘continuous breathing’ is rarely problematic. However, semantic confusion sometimes occurs with animals for whom a ventilation cycle occurs rhythmically but nonetheless only once every several seconds or even every few minutes (amphibians and reptiles), or cetaceans that have an extended inspiratory pause. Should the pauses between these regular breaths be interpreted as end‐inspiratory or end‐expiratory pauses or as some form of apnoea? If the latter, then what is the difference between an inter‐breath interval and a breath‐hold while submerged? With rapid breathing there may be no pause between breaths, but with slower breathing there may be a slight pause, and with very slow breathing the pause may be seconds to minutes. We suggest that if the inter‐breath intervals are relatively regular and repeatable, *and* the pattern is rhythmic, that it be considered continuous (i.e., it is the rhythm that is continuous). Consider as an example the resting breathing pattern of the terrestrial tortoise, *Testudo graeca*. Breaths typically occur regularly at a frequency of 2–3 breaths/min (Figure 2c), and breathing is arrested only when the animal is startled. We contend, in the context of a tortoise with a metabolic rate that is only 10%–20% that of a similarly sized mammal, that despite prolonged intervals between breaths, the tortoise is breathing both regularly and ‘continuously’.

##### Recommendation

We recommend that the terms ‘continuous breathing’ and ‘eupnoea’ be used when breaths immediately follow one another in a rhythmic or arrhythmic fashion, *and* when breaths have pauses between them but are both regular and rhythmic. We recommend this regardless of the length of the pauses between breaths and contrast this with breathing punctuated by irregular periods with breaths and apnoeas of potentially variable length.

#### 3.1.2 ‘Intermittent breathing’

##### The phenomenon

The term ‘intermittent breathing’ is broad and therefore simultaneously useful and ambiguous. As the name suggests, intermittent breathing applies to ventilatory patterns in which breathing is not continuous, that is, not eupnoeic. It applies equally to terrestrial and aquatic animals that ventilate their gills and/or lungs intermittently. Intermittent breathing has been used interchangeably with both ‘periodic breathing’ and ‘episodic breathing’. We consider ‘periodic breathing’ and ‘episodic breathing’ as distinct forms of intermittent breathing arising due to different modulatory influences acting on the central rhythm generator, as described below.

##### Semantic issues

Both the utility and the weakness of ‘intermittent breathing’ lies in its generality. As a general term it is useful in discussions of the consequences of interrupted ventilation of gas exchange. The weakness is that it conveys no information as to mechanism or circumstances and encompasses a variety of patterns. It should be noted that intermittent patterns may still be regular in occurrence and demonstrate an underlying rhythm.

##### Recommendation

**FIGURE 3:**
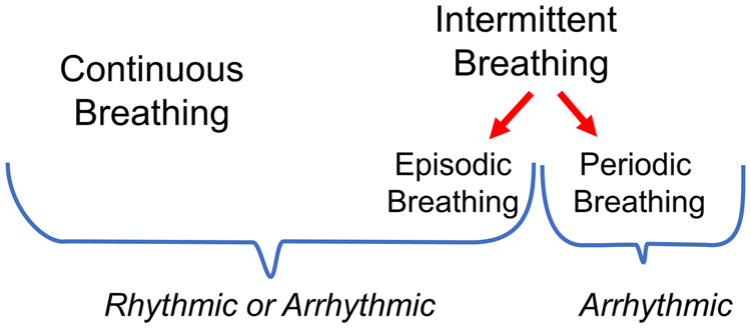
Relationship between different breathing patterns. See text for additional explanation.

As reflected in the title of our review, we advocate the term ‘intermittent breathing’ as an overarching term, with more specific terms being reserved for more specific phenomena associated with either innate or pathological situations (Figure 3).

#### 3.1.3 ‘Periodic breathing’

##### The phenomenon

**FIGURE 4:**
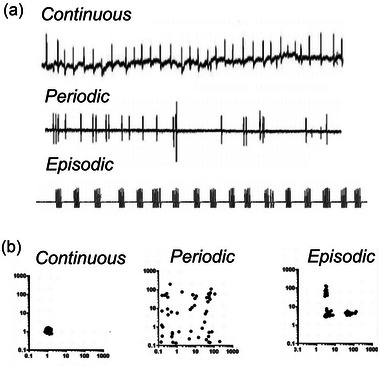
(a) Respiratory traces (each deflection is a breath) illustrating continuous (eupnoeic), periodic and episodic breathing patterns. (b) Poincaré plots of the inter‐breath interval plotted against the following inter‐breath interval for the three different breathing patterns. (W. K. Milsom, unpublished observation.)

In the literature on non‐human species, the term ‘periodic breathing’ implies that there are periods *without active lung ventilation*, that is, that periods of single or multiple breaths are interspersed with distinct periods of apnoea of variable length (Figure 3). Thus, the implied pattern of periodic breathers is a pattern that is neither rhythmic nor continuous when considered over the course of several hours. As such, the term ‘periodic breathing’ encompasses breathing patterns in which single breaths or bursts of breaths occur periodically (Figure 4). We treat as periodic breathing those patterns in which multiple breaths occur in discrete episodes during which lung ventilation may be continuous and rhythmic (see next section). Breathing patterns consisting of isolated single breaths are common in many air‐breathing fish, amphibians and reptiles (Burggren, 1975; Klein et al., 2002; Milsom et al., 2022; Shelton & Boutilier, 1982) as well as in many marine mammals (particularly dolphins and cetaceans, but also pinnipeds) (Andrews et al., 1997; Bickett et al., 2019; Blawas et al., 2021a, 2021b; Castellini et al., 1994; Fahlman, 2024; Fahlman & Madigan, 2016; Fahlman et al., 2020b, 2017, 2023a). Periodic breathing also occurs during hibernation (Malte et al., 2013; Milsom et al., 1999; Sprenger & Milsom, 2022), where it may occur as isolated single breaths, as multiples of breaths or as distinct episodes.

The periodic breathing patterns seen in fish, amphibia and reptiles appear to be an intrinsic property of the central respiratory control system during periods of low respiratory drive. Breathing patterns appear to be the result of an interaction between peripheral sensory inputs and descending central commands. When metabolic rate increases, breathing becomes more regular and may become continuous. The patterns seen in diving reptiles, birds and mammals are also due to similar interactions between peripheral inputs and descending central commands, but because of enhanced O_2_ carrying capacity, they are not restricted to periods of low metabolism. The irregular periods of apnoea seen in these patterns reflect dynamic changes in the balance of modulatory inputs to the CRG.

##### Semantic issues

Unfortunately, in the medical literature the term ‘periodic breathing’ has also been used to describe breathing patterns in which breathing periodically speeds up and/or slows, sometimes with changes in the depth of the breath, often in the context of respiratory control pathologies (Agostoni & Salvioni, 2019; Agostoni et al., 2017; Bader et al., 1996; Randerath et al., 2017). Sometimes, but not always, periodic breathing is accompanied by periods of apnoea. Consequently, in the literature we find three types of periodic breathing: (1) arrhythmic continuous breathing patterns in which breathing speeds and/or slows with changes in breath amplitude, (2) episodic patterns of multiple breath clustered together interspersed with periods of apnoea, and (3) patterns of single breaths or irregular bursts of breaths interspersed with periods of apnoea.

##### Recommendation

We recommend that ‘periodic breathing’ be restricted to the patterns of single breaths or irregular bursts of breaths interspersed with irregular periods of apnoea. Continuous breathing patterns in which breathing speeds and/or slows with changes in breath amplitude we suggest be referred to as arrhythmic forms of continuous breathing as described earlier. The main distinguishing feature between these two breathing patterns is the lack of continuity in the former (periodic) pattern suggesting a significant change in the balance of inputs driving ventilation (Figure 4). We refer to episodic patterns of multiple breaths clustered together interspersed with periods of apnoea as ‘episodic breathing’, which we consider next.

#### 3.1.4 ‘Episodic breathing’

##### The phenomenon

**FIGURE 5:**
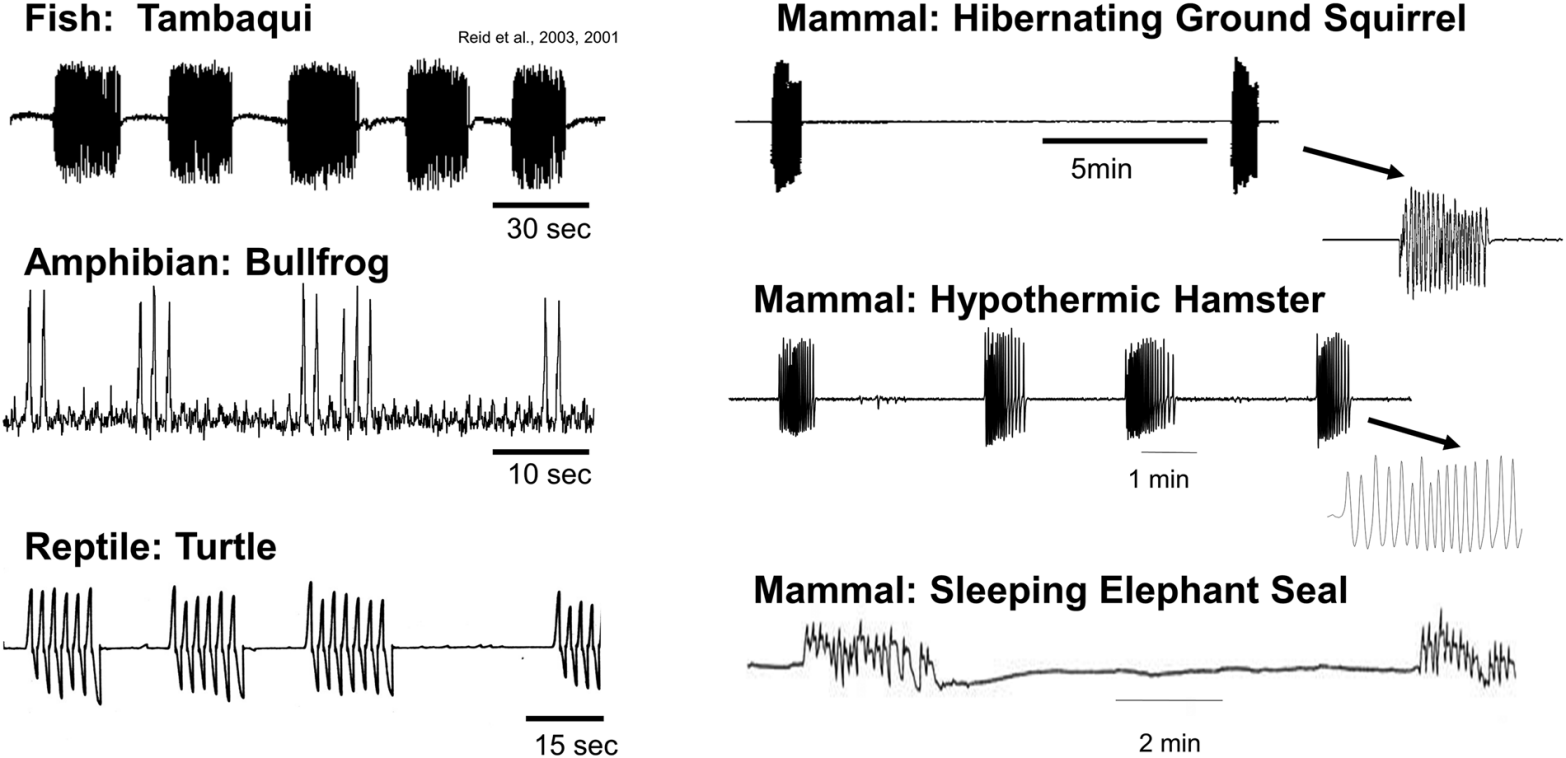
Episodic breathing patterns in fish, reptiles and mammals under different physiological conditions. (W. K. Milsom, unpublished observation.)

Episodic breathing is a form of intermittent breathing in which multiple breaths are clustered into episodes interspersed with periods of apnoea (Figure 3). It can be seen routinely in some species of fish, as well as throughout the Amphibia and Reptilia. Episodic breathing is seen in many, but not all species of heterothermic mammals when in hibernation (Milsom, 1991; Milsom et al., 1997; Nicol & Andersen, 2003), as well as in the late fetuses and neonates of some mammalian species including humans (Champagnat et al., 2009; Rigatto, 1992; Szoták‐Ajtay et al., 2020) (Figure 5). This pattern also appears to be an intrinsic property of the central respiratory control system occurring primarily during periods of low respiratory drive. Removal of descending inputs from the pons in the rostral medulla results in the elimination of the episodes and a pattern of continuous breathing in which the respiratory rhythm is slower than that which was seen in the breathing episodes.

##### Semantic issues

**FIGURE 6:**
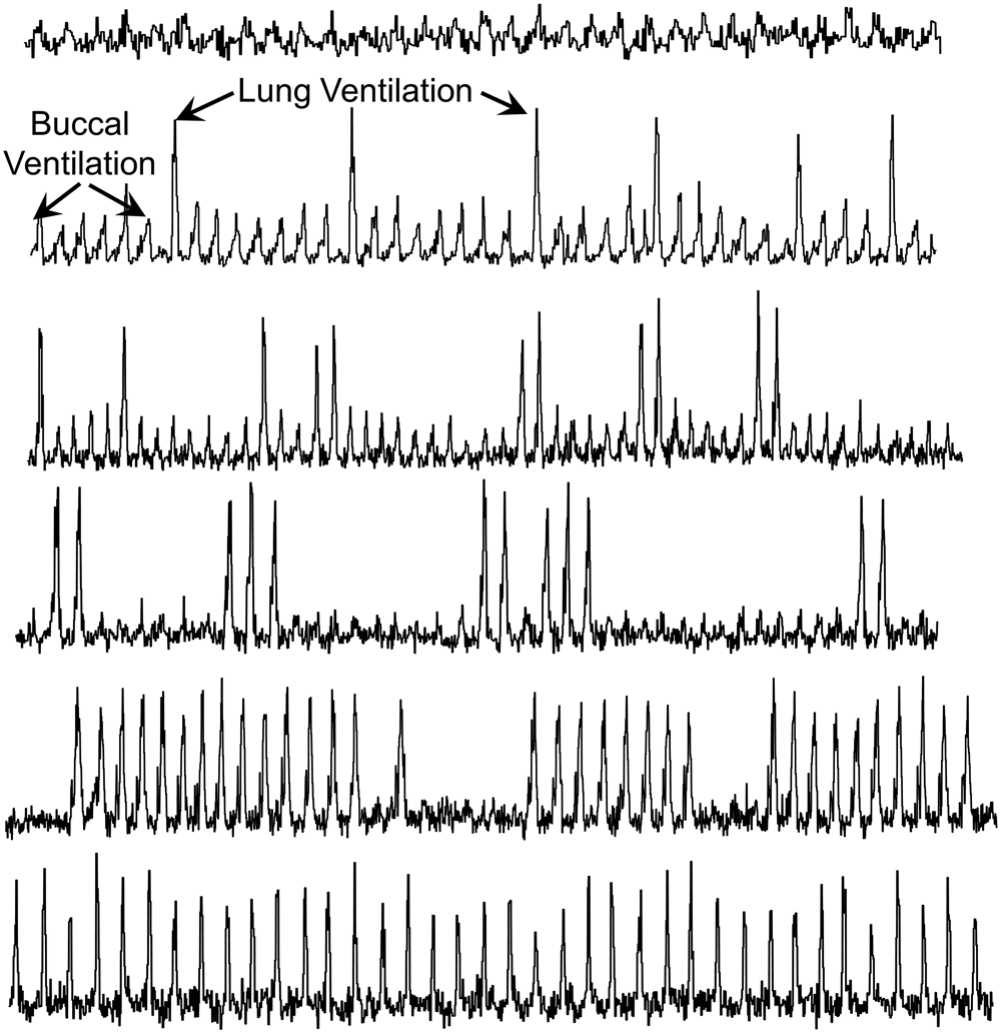
Breathing traces from the frog (*Lithobates pipiens*) breathing increasing levels of CO_2_ (top to bottom) showing single breaths giving rise to breathing episodes giving rise to continuous breathing. (Large deflections are lung breaths, smaller deflections indicate buccal oscillations.) (J. Chatburn and W. K. Milsom, unpublished observation.)

In many of the species exhibiting episodic breathing, increases in respiratory drive will lead to continuous breathing while reduction is respiratory drive may result in fewer and fewer breaths in each episode until only periodic single breaths occur (Figure 6). This begs the question of whether these are episodes of one breath or a change in pattern to one of slow continuous breathing.

**FIGURE 7:**
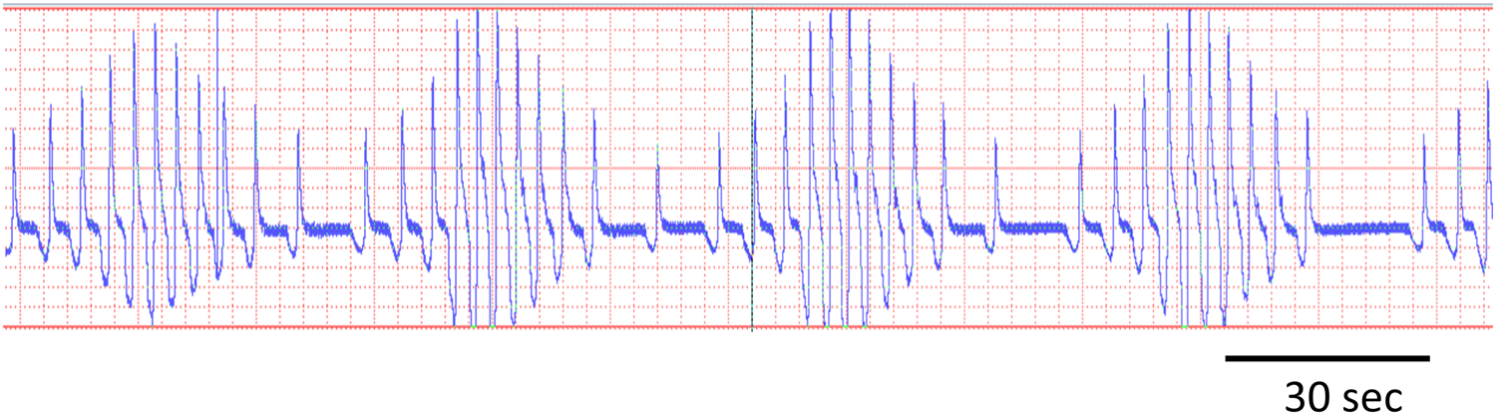
Cheyne–Stokes breathing pattern in a cormorant (*Phalacrocorax pelagicus*). (J. Meir and W. K. Milsom unpublished.)

Some forms of episodic breathing in humans are pathological—for example, Cheyne–Stokes breathing seen in heart failure, with obstructive or central sleep apnoea and at high altitude (Coniglio & Mentz, 2022; Javaheri & Brown, 2017; Mittal et al., 2023). In this pattern there is a waxing and waning of the breaths within each episode (Figure 7). One theory ascribes Cheyne–Stokes respiration to a neurological abnormality, due either to an excessively depressed respiratory centre or to an excessively excitable one. It can also arise due to the prolongation of the circulation time (as in congestive heart failure) or to time delays in the transmission of information and low damping ratios (Cherniack & Longobardo, 1973). Cheyne–Stokes breathing also arises due to the mismatch between the hypoxic drive to breathe and the ensuing hypocapnia due to hyperventilation putting a brake on breathing as seen at altitude (Berssenbrugge et al., 1983). All can lead to instability in feedback control systems. Apnoeas may or may not appear between episodes.

##### Recommendation

We recommend that ‘episodic breathing’ be restricted to situations in which breaths are clustered into discrete episodes with intervening apnoeas. We further recommend that Cheyne–Stokes breathing, with distinct underlying pathological mechanisms, be treated as a distinct pattern of its own.

#### 3.1.5 ‘Discontinuous breathing/ventilation’

##### The phenomenon

‘Discontinuous breathing’ and ‘discontinuous ventilation’ are phrases that have been adopted by insect biologists to describe intermittent ventilation of the tracheal system (Bradley, 2006; Contreras et al., 2014; Matthews & White, 2013; Oladipupo et al., 2022) with attendant ‘discontinuous gas exchange’ (Lighton, 2007). The mechanism that underlies this is an interaction between the need to eliminate CO_2_ while restricting respiratory water loss and controlling reactive oxygen species formation (Bradley, 2006).

##### Semantic issues

‘Discontinuous breathing’ has also been used to describe ventilation patterns in mammals, including humans (Morris et al., 1994), as well as amphibians and other lower vertebrates (Taylor et al., 2014; Winwood‐Smith & White, 2018). The patterns seen in these groups, however, are due to other mechanisms and fit well within the framework described above.

##### Recommendation

Because the term ‘discontinuous breathing’ is so heavily ‘branded’ in the insect physiology community, we recommend that ‘discontinuous ventilation’ be restricted to that taxon (while recognizing that most medical physiologists are unlikely to be aware of the insect literature).

#### 3.1.6 ‘Apnoea’

##### The phenomenon

Definitions of breathing patterns based on the presence or absence of periods of apnoea beg a definition of what constitutes a period of apnoea. Apnoea is typically defined as the temporary cessation of breathing. When metabolic rates are slow, pauses appear in the breathing pattern either at end‐inspiration (amphibians, reptiles, birds and marine mammals) or end‐expiration (terrestrial mammals). Yet, at what point this pause becomes an apnoea is arbitrarily defined in different ways by different researchers. In humans, apnoea has been defined as a non‐ventilatory period of 10–15 s, while in reptiles and amphibians it is defined as a period equal to two or more normal breathing cycles. An advantage of the latter definition is that it will scale with body size since breathing frequency scales with body mass to −0.24 in terrestrial mammals and between −0.34 and −0.42 in aquatic mammals (He et al., 2023; Mortola & Limoges, 2006).

##### Semantic issues

Based simply on its etymology, ‘apnoea’ is agnostic as it ignores the cause of the lack of breathing. Thus, the use of ‘apnoea’ can be problematic if the reader is trying to infer mechanism. Indeed, ‘apnoea’ and ‘apnoeusis’ in the medical literature are typically associated with pathological conditions (e.g., sleep apnoea), whereas apnoeas in diving animals are obviously a normal condition when submerged.

##### Recommendation

Given the preceding discussion of breathing patterns, we suggest that the term ‘apnoea’ be defined as periods of cessation of breathing significantly longer than a normal inter‐breath interval (i.e., end‐expiratory or end‐inspiratory pause), noting that these normal pauses are metabolic rate‐dependent.

#### 3.1.7 ‘Breath‐holding’

##### The phenomenon

A breath‐hold is an apnoea either at end‐inspiration, in amphibians and reptiles and also diving birds (Schell et al., 2023), cetaceans and sea lions (Ponganis, 2011), or following expiration, as in seals (Ponganis, 2011). Breath‐hold following end expiration is accompanied by airway closure and relaxation of the expiratory muscles. As such it is not clear whether the breath‐hold should be considered as part of the inspiratory or early expiratory phase.

##### Semantic issues

‘Breath‐holding’ is simultaneously one of the simplest terms used and one of the most confounding. The lay person certainly understands the concept of breath‐holding, but at the same time this term can convey a cognitive action rather than the simpler cessation of breathing. As we alluded to earlier, a term like ‘breath‐holding’ can imply both a mechanism and cognition.

##### Recommendation

We recommend that ‘breath‐holding’ as a term be employed with caution, recognizing that it has power as a lay term, but also has somewhat restricted meaning to the physiologist. ‘Intermittent breathing’ with periods of apnoea is a more appropriate term when describing an overall ventilatory pattern, while ‘breath‐holding’ can effectively be used to describe a certain state at a certain time such as the procedural instruction to human patients undergoing diagnostic testing (Kravchenko et al., 2023; Ma et al., 2019) or its use in breath‐hold diving in humans (Patrician et al., 2021; Tetzlaff et al., 2021).

### 3.2 Terms used to describe heart beat patterns during intermittent breathing

#### 3.2.1 ‘Respiratory sinus arrhythmia’

##### The phenomenon

**FIGURE 8:**
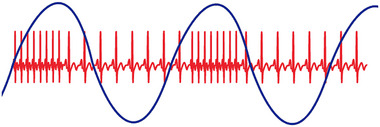
A schematic representation of respiratory sinus arrhythmia (RSA) showing an acceleration of heart rate (ECG) associated with inspiratory air flow and a decrease associated with expiratory air flow. (Reproduced from Fahlman et al., 2020a, 2023a.)

Respiratory sinus arrhythmia (RSA) is classically defined as instantaneous heart rate variation throughout the respiratory phase of ventilation, with acceleration and deceleration of instantaneous heart rate related to the cycle of inspiration and expiration (Figure 8). RSA is a well‐described phenomenon observed across all vertebrate classes (Acharya‐Patel et al., 2018; Angelone & Coulter, 1964; Ben‐Tal et al., 2012; Elstad et al., 2018; Fisher et al., 2022; Hirsch & Bishop, 1981; Lalanza et al., 2023; Melcher, 1976; Taylor et al., 2010, 2022; Yasuma & Hayano, 2004). The rhythmic variation of instantaneous heart rate was first reported over 170 years ago in the dog (Ludwig, 1847). The magnitude of RSA decreases with increasing breathing frequency but increases with increasing tidal volume (Hirsch & Bishop, 1981; Kobayashi, 1998). Both vagal and sympathetic activity appear to modulate RSA (Harms et al., 2013; Taylor et al., 2001). In vertebrates, RSA is thought to improve gas exchange and maintain blood gases by increasing pulmonary flow during the inspiration of fresh air and decrease flow during exhalation (Ben‐Tal et al., 2012; Hirsch & Bishop, 1981; Mortola, 2015; Yasuma & Hayano, 2004). In birds, gas flow is unidirectional through the parabronchi during both inhalation and exhalation of each respiratory cycle (Butler et al., 1988; Cieri & Farmer, 2016; Scheid & Piiper, 1971). We are unaware of any study attempting to investigate whether RSA improves gas exchange in this group.

##### Semantic issues

**FIGURE 9:**
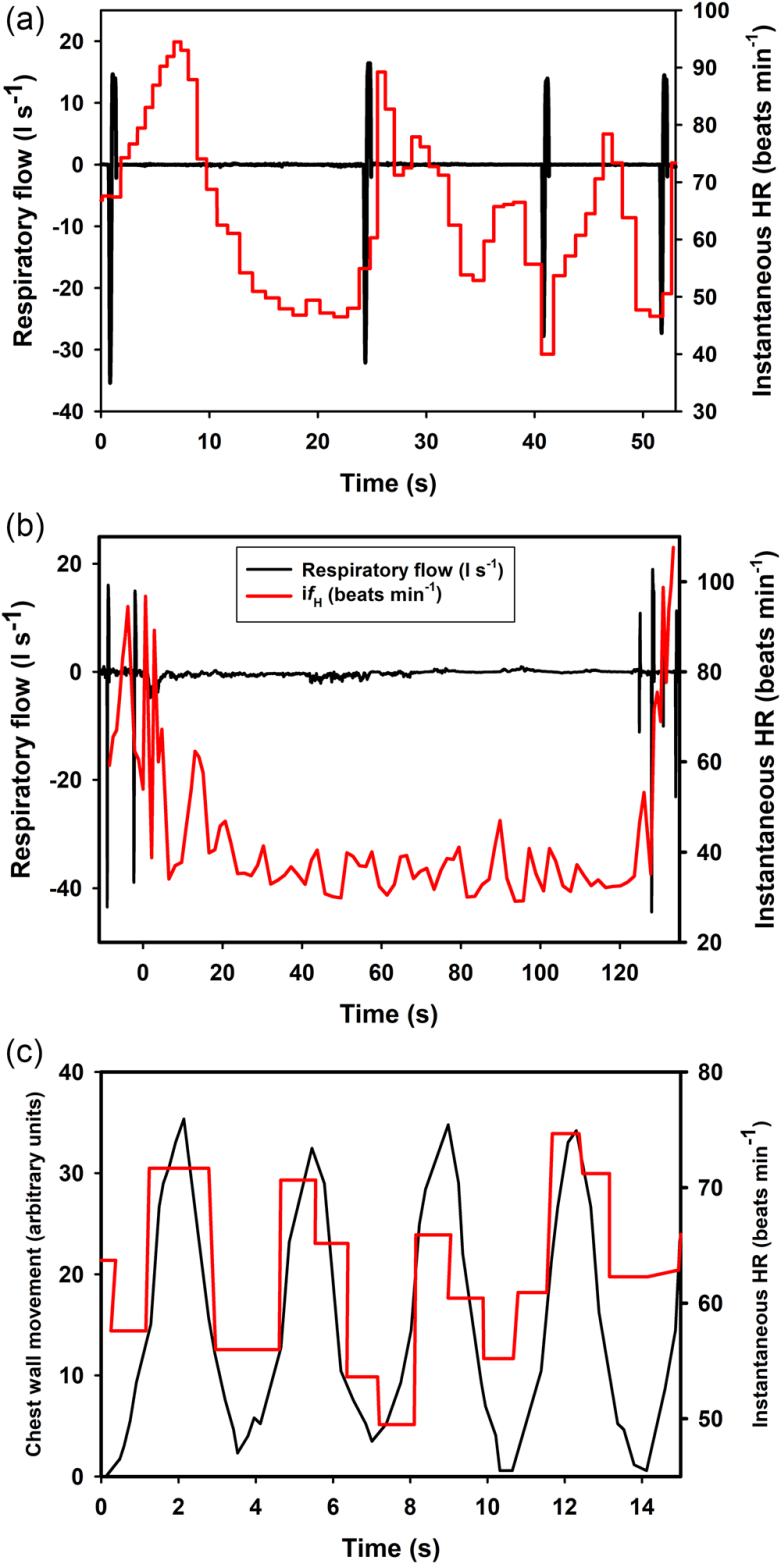
Heart rate changes associated with different types of breathing (a, c) or during a submerged breath‐hold (b). Panels show respiratory flow (l s) or chest wall movement (arbitrary units), and instantaneous heart rate (a) in a dolphin breathing spontaneously (intermittent breathing), (b) immediately before, during and following a breath‐hold, and (c) in a human breathing spontaneously (continuous breathing). ((a) Reproduced from Cauture et al. (2019), (b) from Fahlman et al. (2020a), and (c) from Mortola et al. (2016).)

RSA is classically defined as heart rate variation that occurs with each respiration cycle—specifically acceleration during inhalation and deceleration during exhalation (Fig. 8). This cyclical variation in instantaneous heart rate is the result of cyclical changes in autonomic input to the SA node of the heart driven by the respiratory cycle. Based on misinterpretation of this definition, the variation in heart rate associated with breathing in cetaceans has often been erroneously called RSA (Blawas et al., 2021a, 2021b; Cauture et al., 2019; Elmegaard et al., 2019; Fahlman et al., 2020c, 2019; Harms et al., 2013). However, because in freely diving mammals the breath duration at the surface is only approximately one cardiac cycle, it is impossible to observe any variation in heart rate associated with the expiratory and inspiratory phases as required by the classical definition. The increase in heart rate may begin before or following the breath and the subsequent decrease seconds to minutes later (Figure 9). In elephant seal pups while resting or sleeping on land when breaths are much slower, a respiratory sinus arrythmia has been reported that follows the original definition (see Figure 9 in Castellini et al., 1994). During sleep, seal pups breathe intermittently in episodes. There is a large increase in heart rate associated with the episode (which is not RSA) but small breath by breath oscillations in instantaneous heart rate are superimposed on this. The magnitude of these changes appears to vary with respiratory effort (Castellini et al., 1994; Cauture et al., 2019).

##### Recommendation

We propose that use of the term ‘respiratory sinus arrhythmia’ be confined to cyclical changes in instantaneous heart rate associated with single breaths and that it be discontinued as a description of the cardiovascular changes associated with breathing in cetaceans. In the latter case we propose use of the term ‘ventilation tachycardia’, described below.

#### 3.2.2 ‘Diving bradycardia’

##### The phenomenon

Cardiac slowing during diving has been well studied from its initial description in 1786 by Edmund Goodwyn (1786), subsequently confirmed nearly a century later by Paul Bert (1870), who is typically credited, erroneously, with the first observation of this phenomenon (Vega, 2017). Currently, diving bradycardia is known to vary in its magnitude across taxa, and to be modulated by a number of physiological, environmental and cognitive factors (Blix, 2018; Fahlman, 2024; Lundgren & Miller, 1999; Panneton & Gan, 2020). In his seminal work, Scholander (1940) showed that the reduction in heart rate during diving was accompanied by increased peripheral vasoconstriction, resulting in the maintenance of mean arterial blood pressure. The peripheral vasoconstriction selectively produced a reduction in peripheral blood flow while maintaining perfusion to the heart and brain. Scholander (1963) later proposed that this ‘dive response’ was the ‘master switch’ of life for diving mammals, comprising a vital adaptation that allowed these animals to perform extended dives in pursuit of food. Mottishaw et al. disputed the idea that the reduction in heart rate associated with diving was an adaptation specifically developed for diving, and suggested that it is an ancient response to manage hypoxia (Mottishaw et al., 1999). Currently, diving bradycardia is generally considered to be a reflex response, although in marine mammals it can be conditioned by the planned length or depth of a dive, implicating a role for cognitive processes in the manipulation of heart rate (Elmegaard et al., 2016; Elsner et al., 1966; Fahlman et al., 2020a; Jones et al., 1973; Kaczmarek et al., 2018; Ridgway et al., 1975). Thus, the reduction in heart rate in diving does not appear to be solely an autonomic reflex in the traditional sense.

##### Semantic issues

Whether there is a bradycardia during diving, depends on what heart rates are being compared, since bradycardia, like the term tachycardia, is relative to some baseline heart rate. If one assumes that the baseline heart rate is that which supports a standard metabolic rate, an interesting paradox arises. For species that dive sporadically, foraging for food or to escape predators, with a fall in heart rate associated with a suspension of many physiological processes, then the term ‘diving bradycardia’ seems appropriate. However, for species such as cetaceans, that spend the vast majority of their lives underwater, their diving heart rate is the one that supports their metabolic needs and the elevation in heart rate seen on surfacing to breathe could actually be considered to be a tachycardia enhancing gas exchange. Furthermore, in reptiles, birds and mammals several studies showed that there was no diving bradycardia when dives were shorter than their aerobic capacity (Belkin, 1964; Gaunt & Gans, 1969; Kanwisher et al., 1981; Kooyman, 1985; Kooyman & Campbell, 1972; Lin et al., 1972), though typically diving bradycardia does occur in most dives of most reptiles—for example (Axelsson & Franklin, 2011; Jacob & McDonald, 1976; Joyce et al., 2018; Saito et al., 2022a).

##### Recommendation

We urge authors to carefully consider which of the observed heart rates are ‘baseline’ when making comparisons and using the term ‘bradycardia’. We strongly recommend only using ‘bradycardia’ when heart rates are reduced below the level required to maintain standard metabolic rates. We suggest also reporting the breathing frequency, due to its confounding effect on heart rate and the estimated metabolic rate (Fahlman, 2024; Fahlman et al., 2023b).

#### 3.2.3 ‘Ventilation tachycardia’

##### The phenomenon

As just discussed, a key question when determining terminology is, What is normal? Does the ‘normal’ heart rate occur during air breathing (an anthropocentric perspective), or during diving/breath‐holding? In turtles, breaths are associated with an increase in heart rate, and pharmacological intervention has shown that this ventilatory tachycardia is mediated by a lessening of the inhibitory vagal tone imposed during diving (Burggren, 1975; Gordos et al., 2006; Saito et al., 2022b). During intermittent breathing in the turtle—whether associated with diving or simply with breath‐holding at the water's surface—heart rate increases and remains elevated throughout the ventilation period, but then decreases towards a stable value between bouts of ventilation (Burggren, 1975; Butler et al., 1984; Gordos et al., 2006). The overall increase in heart rate during episodic breathing is greater as compared with during periodic breathing. The ventilatory tachycardia during intermittent breathing in the turtle is similar to that reported in toothed whales, for example, bottlenose dolphin, pilot whale, beluga, killer whale and false killer whale (Blawas et al., 2021b; Cauture et al., 2019). Although the absolute change in heart rate in the odontocete during breathing is greater than that seen in the turtle, the relative change appears to be similar with a doubling in instantaneous heart rate associated with the breath. In the elephant seal pup, episodic breathing resulted in a similar doubling of the instantaneous heart rate, which was maintained throughout the respiratory burst (Castellini et al., 1994).

##### Semantic issues

As mentioned above, the terms bradycardia and tachycardia are relative—there must be a reference heart rate for comparison. In animals that spend most of their time apnoeic or breath‐holding, ventilation tachycardia, as opposed to diving bradycardia, more accurately describes their physiological state.

##### Recommendation

While it seems heretical to do so, again we urge authors to carefully consider which heart rate is baseline when making comparisons. We strongly recommend only using the term ‘diving bradycardia’ when heart rates are reduced below the level required to maintain standard metabolic rates (implying increased vagal tone) and using the term ‘ventilation tachycardia’ when heart rates increase with intermittent breathing to rates above those required to sustain normal metabolic rates (implying a reduction in vagal tone).

## 4 CONCLUSIONS AND RECOMMENDATIONS

### 4.1 The semantic problem

As Alfred Korzybiski, the father of modern semantics, commented, ‘Whatever you say it is, it isn't’. As we have reviewed above, we see a semantic conflict, or at least confusion, in the variety of terms used to describe phenomena—and often the same phenomenon—associated with intermittent breathing and/or diving. Ultimately, however, we emphasize that the biology behind the phenomenon is most important, not what we call it. Yet, as mentioned in our introduction, physiological mechanisms often lurk behind the terms we use. To call out use of the term ‘diving bradycardia’ as an example, this suggests to some an (ab)normal decline in heart rate during diving when, in fact, for many diving animals most of their lives may be spent not breathing rather than breathing.

### 4.2 Recommendations

When we embarked on this project, we naively (and arrogantly?) had a goal of ‘setting the record straight’ regarding terminology. However, as we began to document the myriad terms used in the field of diving biology, generally writ, as well as the surprising degree of passion that we encountered in our informal semantic discussions with biologists in the field of diving physiology, we recognized that the ‘terminology genie’ may very well be out of the bottle, with no putting it back.

**FIGURE 10:**
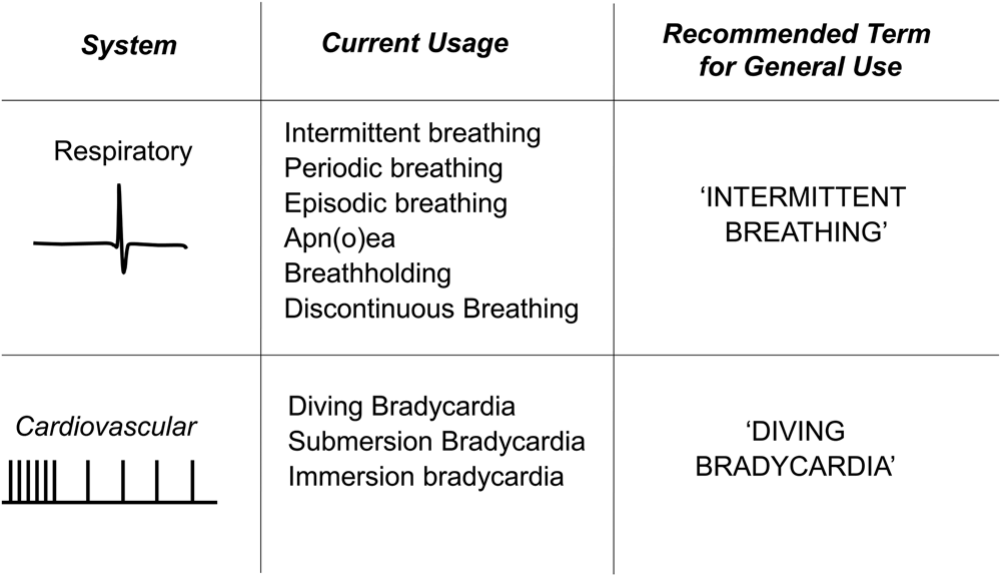
Recommended terms for general use when describing respiratory and cardiac phenomena associated with intermittent breathing. Note that the term diving bradycardia is recommended only if heart rate falls below that associated with standard metabolic rate.

Having said that, we suggest generic terms *when applied to animals that normally show periods when they cease air breathing*, whether they are submerging in water or not (Figure 10). As is often the case, context is all‐important, but we do strongly advise against using any term associated with human pathologies to describe ventilation and circulatory patterns in normally behaving, intermittently breathing animals. With respect to ventilation of an air‐breathing organ, we suggest the generic term ‘intermittent breathing’ be used when feasible, and the terms ‘apnoea’, ‘periodic breathing’ and ‘episodic breathing’ be used only when appropriate. When limited to a single word with utility, more difficult is finding a benign description of the associated cardiac changes accompanying intermittent breathing. We recommend that careful thought be given to what normal heart rate is and when it occurs when using the terms ‘bradycardia’ and ‘tachycardia’.

Having earlier acknowledged the improbability of convergence on a single set of ‘acceptable’ terms for intermittent breathing and the associated changes in heart rate, we do recommend that biologists be both thoughtful and deliberate in their use of terminology. Consider Blaise Pascal's comment, ‘Words differently arranged have a different meaning, and meanings differently arranged have different effects’ (Pascal, 1670).

## AUTHOR CONTRIBUTIONS

All authors were involved in the writing of the paper and approved the final submission. Author order is alphabetical. All authors have read and approved the final version of this manuscript and agree to be accountable for all aspects of the work in ensuring that questions related to the accuracy or integrity of any part of the work are appropriately investigated and resolved. All persons designated as authors qualify for authorship, and all those who qualify for authorship are listed.

## CONFLICT OF INTEREST

The authors declare no conflicts of interest.
